# Maternal concern and perception about child overweight risk, maternal feeding practices, and child eating behaviors: A cross-sectional study

**DOI:** 10.1371/journal.pone.0302557

**Published:** 2024-06-11

**Authors:** Hebah Alawi Kutbi, Rana Hisham Mosli

**Affiliations:** Department of Clinical Nutrition, Faculty of Applied Medical Sciences, King Abdulaziz University, Jeddah, Saudi Arabia; Al-Ahliyya Amman University, JORDAN

## Abstract

Abundant efforts have been directed to understand the global obesity epidemic and related obesogenic behaviors. However, the relationships of maternal concern and perception about child weight with child eating behaviors in Saudi Arabia have not been investigated. Therefore, this study aimed to examine the associations of maternal concern and perception about child overweight risk with maternal feeding practices and child eating behaviors among mothers and their children in Saudi Arabia. Mothers of 115 children aged 3–5 years old were recruited from eight preschools. Child eating behaviors (enjoyment of food, responsiveness to foods, food fussiness, satiety responsiveness, and slowness in eating) and maternal feeding practices (restriction, monitoring, and using food as a reward), maternal concern about child weight, and maternal perception about child overweight risk were assessed using validated questionnaires. Multiple linear regression models, adjusted for maternal body mass index (BMI) and child’s BMI-z score and sex, were tested to examine the independent associations of maternal concern and perception about child overweight risk with child eating behaviors and maternal feeding practices. Maternal concern about child weight was positively associated with food enjoyment in children (B = 0.14 [95% confidence intervals = 0.02,0.27]) and responsiveness to food (B = 0.16 [0.03,0.29]), whereas a negative association with child food fussiness was observed (B = -0.19 [-0.33, -0.04]). Maternal perception about child overweight risk and child slowness in eating were negatively associated (B = -0.72 [-0.16 to -0.28]). Maternal concern and perception about child overweight risk were not associated with maternal feeding practices. In conclusion, maternal concern and perception about child overweight risk were found to be associated with food approach eating behavior traits but not with maternal feeding practices. Mothers who are concerned about child weight or perceive their children as overweight may need guidance and support to promote healthy eating behaviors among their children. Future cross-cultural studies are needed to understand the underlying mechanisms for the influence of maternal concern and perception about child overweight risk on maternal feeding practices, child eating behaviors, and weight status of children.

## Introduction

Childhood overweight and obesity is a public health burden affecting 38 million children around the world [1]. Hence, abundant efforts have been directed to understand the global obesity epidemic and related obesogenic behaviors to develop effective interventions [2–5]. The current literature indicates that excess adiposity among children is strongly linked to eating behaviors. For example, children exhibiting high food responsiveness, defined as eating in response to food cues, or low ability to adjust eating in response to satiety or fullness are more vulnerable to overweight or obesity [6]. Increased eating speed has been also proposed to increase children’s risk for overweight or obesity [7]. However, children’s eating behaviors (CEBs) are more likely to be influenced by extrinsic factors, such as the home environment [3].

Parents are often recognized to have a powerful influence in shaping eating behaviors of their children through parenting and feeding practices [5]. However, the existing data suggest that parental use of particular feeding practices might be influenced by CEBs or weight status of children [8]. Feeding practices could be also affected by parental concern and perception about child’s overweight risk [9–13]; Parents concerned about their child being overweight or obese may alter the employed feeding practices in order to mitigate unexpected weight gain [14]. However, and in contrast to their intention, the adopted feeding practices may unintentionally promote obesity traits [15, 16].

The impact of maternal weight perception and concern on child’s feeding practices has been previously investigated in a variety of settings and findings varied across cultures. For instance, Brodribb et al. examined this relationship among mothers and infants residing in Queensland; Maternal infant weight perception and concern were found positively correlated with controlling feeding practices [9]. Similar findings have been reported among children aged 5–8 years [10–13]. On the other hand, a study conducted among low-income Latina mother-child dyads did not detect an association between maternal perception and concern about child weight and maternal feeding practices [17], whereas in Saudi Arabia, maternal concern and perception about child weight and the relationship with maternal feeding practices and CEBs have not been previously explored.

Given the established associations of maternal feeding practices and CEBs with children’s body weight status [18], we aimed to build on existing work by examining the relationships of maternal concern and perception about child overweight risk with maternal feeding practices and child eating behaviors among Saudi preschoolers and their mothers. In Saudi Arabia, mothers are often recognized to have greater role and responsibility in child feeding compared to other cultures, which may make them feel accountable for children’s weight status [19, 20]. Additionally, it has been estimated that approximately one-third of the Saudi children (27.8%) are overweight or obese [21]. Therefore, findings of the present study will inform childhood obesity interventions targeting mothers and CEBs.

## Materials and methods

### Study sample and procedure

Data of this study were collected between October and December 2019 from eight preschools; two preschools located in each of the Northern, Southern, Eastern, and Western areas of Jeddah city, Saudi Arabia. Permission to collect data from each school was obtained from school principals. All children enrolled in these schools had an envelope placed in their backpacks, which is routinely checked by their mothers on a daily basis. Each envelope contained an invitation letter for the mother describing the study objectives and protocol and a consent form for participation. The inclusion criteria included that the mother is an Arabic speaker, Saudi or a permeant resident in Saudi Arabia, has a healthy child aged 3–5 years without serious health condition or food allergy, and lives primarily with the child.

The minimum sample size needed for the present size was 113 mothers of preschoolers, based on an estimated correlation of 0.30 between maternal concern about child weight and CEBs, alpha (two-sided) = 0.05, and power of 90% [22, 23]. Mothers who met the inclusion criteria and dispatched the signed consent forms with their children (n = 209) were contacted over the phone to complete the study questionnaire. Ninety-four mothers did not respond to the phone calls over a period of one-month (45.0%); therefore, the final analyses included data of 115 mothers and their children (55.0%).

The study questionnaire assessed the sociodemographic characteristics of the sample, CEBs, and maternal feeding practices, wherein the research assistants read each question aloud and recorded mothers’ response. Child anthropometric measurements were objectively assessed at the schools by trained research assistants. This study was performed in line with the principles of the Declaration of Helsinki. Ethical approval was granted by the Unit of Biomedical Ethics at King Abdulaziz University Hospital (Reference number HA-02-J-008).

### Measures

#### Sociodemographic variables and anthropometric measures

Sociodemographic data (children’s date of birth, nationality, household income, maternal education, and employment status) were collected through phone interviews. Mothers were also requested to recall and report their height and weight, and maternal body mass index (BMI) was calculated. Mothers reporting pregnancy at the time of data collection were asked to provide their pre-pregnancy weight and the BMI was calculated accordingly.

Anthropometric measurements of each child (height and weight) were assessed objectively by trained research assistants within one-week of the conducted phone interview. Heavy clothes and shoes were removed, and measurements were taken twice. Each child was requested to stand still to measure his/her height and if the two readings differed by more than 0.5 cm two additional measurements were taken and the average was calculated. A digital scale was used to measure child’s weight; If weight measurement varied by more than 0.1 kg the weight was reassessed two additional times and the average was recorded. The BMI-z scores for children were calculated according to the reference standards of the World Health Organization (WHO) [24] and used as a continuous variable in all models.

#### Child eating behaviors

CEBs were assessed using five constructs adapted from the Child’s Eating Behaviors Questionnaire (CEBQ), including *enjoyment of food* (desire to eat and interest in food):“My child loves food,” “My child is interested in food,” “My child looks forward to mealtimes,” and “My child enjoys eating”; *food responsiveness* (eating in response to food cues): “My child is always asking for food,” “If allowed to, my child would eat too much,” “Given the choice, my child would eat most of the time,” “Even if my child is full up s/he finds room to eat his/her favorite food,” and “If given the chance, my child would always have food in his/her mouth”; *satiety responsiveness* (ability to adjust eating in response to satiety or fullness): “My child has a big appetite” (reverse coded), “My child leaves food on his/her plate at the end of a meal,” “My child gets full before his/her meal is finished,” “My child gets full up easily,” and “My child cannot eat a meal if s/he has had a snack just before”; *food fussiness* (selectivity in accepting a range of foods): “My child refuses new foods at first,” “My child enjoys tasting new foods” (reverse coded), “My child enjoys a wide variety of foods” (reverse coded), “My child is difficult to please with meals,” “My child is interested in tasting food s/he hasn’t tasted before” (reverse coded), and “My child decides that s/he doesn’t like a food, even without tasting it”; and *slowness in eating* (a slow eating rate): “My child finishes his/her meal quickly” (reverse coded), “My child eats slowly,” “My child takes more than 30 minutes to finish a meal,” and “My child eats more and more slowly during the course of a meal” [25]. Response options for each item ranged from 1 = never to 5 = always. The average score for the items included in each construct was calculated to indicate the average score. These constructs have been widely used and were validated among children in Saudi Arabia [26]. The constructs showed acceptable internal consistency among the sample, wherein Cronbach’s alpha coefficients ranged between 0.61 to 0.80.

#### Maternal concern and perception about child overweight risk

Maternal concern and perception about child overweight risk were assessed using the two constructs of the Child Feeding Questionnaire-Arabic version (CFQ-A), which has been previously validated among preschoolers in Saudi Arabia [20, 27]. *Maternal concern about child weight* was evaluated using three questions: “How concerned are you about your child eating too much when you are not around her?” “How concerned are you about your child having to diet to maintain a desirable weight?” and “How concerned are you about your child becoming overweight?” Response options of each item ranged between “1 = unconcerned” to “5 = very concerned”. *Maternal perception about child overweight risk* was evaluated using the perceived child weight construct (four items) that assessed the perception of child weight status history: “Your child during the first year of life,” “Your child as a toddler,” “Your child as a preschooler,” and “Your child kindergarten.” Response options of each item were as follows: “1 = markedly underweight”, “2 = underweight”, “3 = normal”, “4 = overweight”, and “5 = markedly overweight”. The two constructs showed high internal reliability, of which Cronbach’s alpha coefficients were 0.86 and 0.73, respectively.

#### Maternal feeding practices

Three maternal feeding practices were assessed using constructs obtained from CFQ-A [20], including maternal *restriction* (“I have to be sure that my child does not eat too much of her favorite food,” “I intentionally keep some foods out of my child’s reach,” “If I did not guide or regulate my child’s eating, she would eat too many junk foods,” and “If I did not guide or regulate my child’s eating, she would eat too much of her favorite foods,” response options ranged from “1 = disagree” to “5 = agree”, α = 0.66); *using food as a reward* (“I give my child food to keep him/her quiet when shopping or traveling,” “I give my child food to reward him/her for good behavior,” “I withhold a food my child likes as a consequence for bad behavior,” “My child expects to be given a favorite food as a reward,” “I give my child a special food to celebrate an achievement,” and “I give my child food to persuade him/her to do something he/she does not really want to do,” with response options ranging from “1 = never” to “5 = very often”, α = 0.82); and *monitoring* (“How much do you keep track of the sweets (candy, ice cream cake, pies, pastries) that your child eats?” “How much do you keep track of the snack food (potato chips, Doritos, cheese puffs) that your child eats?” and “How much do you keep track of the high-fat foods that your child eats?” response options ranged from “1 = never” to “5 = always”, α = 0.83).

### Statistical analysis

Data were described as mean ± standard deviation and frequency and percentages. Shapiro-Wilk test of normality was conducted to evaluate normality of continuous data. The results indicated that data were skewed (P < 0.05); therefore non-parametric tests were used. The correlations between the continuous variables were tested using Spearman’s correlation test. Differences in maternal concern and perception about child overweight risk across the groups of categorical variables were assessed using Mann-Whitney and Kruskal-Wallis tests. Multiple linear regression analyses (adjusted for child’s BMI-z score and sex and maternal BMI) were conducted to examine the independent associations of maternal concern and perception about child overweight risk with maternal feeding practices (restriction, using food as a reward, monitoring) and CEBs (food fussiness, slowness in eating, food enjoyment and responsiveness, and satiety responsiveness). We used two-sided tests for the analyses, and *P* value < 0.05 was set to indicate significance. All the analyses were performed using Statistical Packages for Social Sciences (SPSS) version 24.0 (Armonk, NY).

## Results

### Characteristics of the sample and maternal concern and perception about child overweight risk

Table 1 shows the sociodemographic characteristics of the participants. Approximately two-thirds of the children were boys (n = 68, 59.1%). The mean BMI-z score of children was 0.21±1.38, ranging between -3.64 and 4.37. The majority of children in our sample were Saudis (n = 86, 74.8%). Maternal age ranged between 20.1 to 50.1 years old. Approximately half of the mothers were unemployed (n = 62, 53.9%), whereas the majority had completed a college degree or higher (n = 92, 80%). Maternal BMI was found to be positively correlated with the BMI-z score of children (r = 0.26, *P* = 0.01).

**Table 1:** Sociodemographic characteristics of preschool-aged children and their mothers.

Sociodemographic variables		n = 115
Sex, n (%)	Girls	47 (40.9)
	Boys	68 (59.1)
Age, mean (SD)	4.91 (0.78)	
BMI-z score, mean (SD)	0.21 (1.38)	
Saudi nationality, n (%)	Yes	86 (74.8)
	No	29 (25.2)
Maternal age, mean (SD)	33.3 (5.04)	
Maternal working status, n (%)	Employed	53 (46.1)
	Unemployed	62 (53.9)
Household income in SAR, n (%)	< 5000	11 (0.90)
	5000–10000	41 (35.7)
	>10000	63 (54.8)
Maternal education status, n (%)	High school or less	23 (20.0)
	College degree	79 (68.7)
	Post-graduate degree	13 (11.3)
Maternal BMI, mean (SD)	26.7 (5.10)	

Abbreviations: SD, standard deviation; BMI, body mass index; SAR, Saudi Arabian Riyals.

The mean scale scores of maternal concern and perception about child overweight risk and CEBs are illustrated in Table 2. Bivariate analyses were also conducted to investigate whether maternal concern and perception about child overweight risk are linked to the sociodemographic variables. Significantly positive but weak correlation was observed between maternal concern and maternal BMI (r = 0.19, *P* = 0.04). The mean scale scores of maternal concern and perception about child overweight risk were not found to be significantly different across all the sociodemographic groups (P > 0.05).

**Table 2:** Mean scale scores and standard deviations for maternal concern and perception about child overweight risk, feeding practices, and child eating behaviors, from a possible range of 1–5.

Scale	Mean	SD
***Maternal variables***		
Concern about child weight	1.56	1.09
Perception about child overweight risk	2.90	0.37
Use of restriction	3.53	1.20
Using food as a reward	2.53	1.07
Use of monitoring	4.43	0.78
***Child eating behavior***		
Enjoyment of food	2.82	0.74
Food responsiveness	1.93	0.76
Satiety responsiveness	3.59	0.70
Food fussiness	3.30	0.84
Slowness in eating	3.30	0.77

### The correlation between maternal concern and perception about child overweight risk, maternal feeding practices, and child eating behaviors

Table 3 illustrates the correlation between maternal concern and perception about child overweight risk, maternal feeding practices, and CEBs. No correlation between maternal concern about child weight and maternal use of restriction (r = 0.05), using food as a reward (r = -0.09), or monitoring (r = -0.14) was observed (*P* > 0.05 for all). Yet, maternal concern about child weight was positively correlated with child enjoyment of food (r = 0.25, *P* < 0.01) and child responsiveness to food (r = 0.21, *P* = 0.02).

**Table 3:** Spearman’s correlation matrix of maternal concern and perception about child overweight risk, maternal feeding practices, child body mass index z-score, and child eating behaviors (n = 115).

Variables	Maternal variables		Child variables					
	Concern about child weight	Perception about child overweight risk	Body mass index z-score	Enjoyment of food	Food responsiveness	Satiety responsiveness	Food fussiness	Slowness in eating
***Maternal variables***								
Concern towards child weight	1.00	0.07	-0.02	0.25**	0.21*	-0.04	-0.15	-0.01
Perceived child weight	0.07	1.00	0.48**	0.14	0.01	-0.04	-0.07	-0.34**
Use of restriction	0.05	0.12	0.06	0.17	0.08	0.16	0.02	0.04
Using food as a reward	-0.09	-0.04	0.04	0.04	0.17	0.10	0.05	0.10
Use of monitoring	-0.14	0.10	0.14	-0.20*	-0.29**	0.00	-0.02	-0.13
***Child variables***								
Body mass index z-score	-0.02	0.48**	1.00	--	--	--	--	--
Enjoyment of food	0.25**	0.14	-0.01	1.00	--	--	--	--
Food responsiveness	-0.01	0.01	-0.01	0.54**	1.00	--	--	--
Satiety responsiveness	-0.04	-0.04	-0.06	-0.44**	-0.22*	1.00	--	--
Food fussiness	-0.15	-0.07	-0.02	-0.22*	0.02	0.34**	1.00	--
Slowness in eating	-0.01	-0.34**	-0.19*	-0.04	0.12	0.15	0.29**	1.00

* *P* < 0.05.

** *P* ≤ 0.01.

Maternal perception about child overweight risk was not found to be correlated with maternal feeding practices, including use of restriction (r = 0.12), using food as a reward (r = -0.04), or monitoring (r = 0.10), (*P* > 0.05 for all). However, maternal perception about child overweight risk was found to be positively correlated with child’s BMI-z score (r = 0.48, *P* < 0.01), whereas a negative significant correlation was observed with child slowness in eating (r = -0.34, *P* < 0.01).

### Associations of maternal concern and perception about child overweight risk with maternal feeding practices

Multiple regression analyses were conducted to investigate whether maternal concern and perception about child overweight risk predict maternal feeding practices and CEBs, Table 4. Maternal concern was not associated with the use of restriction (B = 0.17 [95% confidence intervals (CI) = -0.05,0.37]), using food as a reward (B = -0.11 [-0.30,0.08]), or monitoring (B = -0.04 [-0.18, 0.10]). Similarly, maternal perception was not found to be associated with maternal use of restriction (B = 0.60 [-0.11, 1.31]), using food as a reward (B = 0.45 [-0.18, 1.09]), or monitoring (B = -0.14 [-0.61, 0.32]).

**Table 4:** Multiple regression analyses predicting maternal feeding practices and child’s eating behaviors ^a^.

Dependent variable	Independent variables									
	*Maternal concern about child’s body weight*					*Maternal perception about child’s body weight*				
	R2	Beta	SE	*P* value	95% CI	R2	Beta	SE	*P* value	95% CI
***Maternal feeding practices***										
Use of restriction	0.04	0.17	0.11	0.12	-0.05, 0.37	0.04	0.60	0.39	0.10	-0.11, 1.31
Using food as a reward	0.05	-0.11	0.09	0.25	-0.30, 0.08	0.05	0.45	0.32	0.16	-0.18, 1.09
Use of monitoring	0.03	-0.04	0.07	0.58	-0.18, 0.10	0.03	-0.14	0.23	0.54	-0.61, 0.32
***Child’s eating behaviors***										
Enjoyment of food	0.07	0.14	0.06	0.03	0.02, 0.27	0.05	0.32	0.22	0.15	-0.12, 0.76
Food responsiveness	0.06	0.16	0.07	0.02	0.03, 0.29	0.01	0.10	0.23	0.67	-0.36, 0.55
Satiety responsiveness	0.01	-0.04	0.06	0.52	-0.16, 0.08	0.02	0.26	0.21	0.22	-0.16, 0.68
Food fussiness	0.06	-0.19	0.07	0.01	-0.33, -0.04	0.02	-0.30	0.25	0.24	-0.80, 0.20
Slowness in eating	0.03	-0.02	0.07	0.76	-0.16, 0.11	0.11	-0.72	0.22	0.002	-1.16, -0.28

Abbreviations: SE, standard error; CI, confidence interval.

^a^ Covariates include maternal body mass index, child’s body mass index z-score, and child’s sex.

### Associations of maternal concern and perception about child overweight risk with child eating behaviors

Positive associations were observed between maternal concern about child weight and child enjoyment of food (B = 0.14 [0.02,0.27]) and responsiveness to food (B = 0.16 [0.03, 0.29]), whereas a negative association with child’s food fussiness was found (B = -0.19 [-0.33, -0.04]). Maternal perception about child overweight risk was inversely related to child slowness in eating (B = -0.72 [-0.16, -0.28]).

## Discussion

The present study was the first to examine the relationships of maternal concern and perception about child overweight risk with maternal feeding practices and CEBs in Saudi Arabia. Contrary to findings of other studies [10, 13], maternal concern and perception about child overweight risk were not found to be associated with maternal feeding practices among our sample. On the other hand, maternal concern about child weight predicted higher levels of child enjoyment and responsiveness to food and lower levels of food fussiness, whereas maternal perception about child overweight risk was negatively associated with child slowness in eating.

Children are born with an innate ability to regulate their food intake. However, as they grow, their eating behaviors are increasingly influenced by extrinsic factors such as parental eating behaviors, foods made available to children, and the utilized feeding styles [28]. The existing evidence indicates that CEBs are strongly related to their weight status. Overweight children tend to be less responsive to satiety clues and more responsive to food compared to normal-weight children, whereas low responsiveness to satiety clues has been linked to child’s weight gain [29]. Hence, efforts to reduce the prevalence of childhood overweight and obesity had focused on understanding CEBs and associated factors that could influence their weight status. Nevertheless, findings of the present study suggest that maternal concern and perception about child overweight risk is an important area to consider when designing childhood obesity interventions.

Even though children’s body weight in the present study was not found to be associated with maternal concern about child weight, the higher degree of concern about child weight predicted greater food enjoyment and responsiveness in children and lower levels of food fussiness. Food enjoyment and responsiveness are food approach eating behavior traits that have been linked to increased child weight [30], whereas food fussiness is a food avoidance eating behavior trait that has been linked to decreased child weight [30]. These findings indicate that maternal concern about child weight could unintentionally result in counterproductive effect promoting obesity traits. Several studies have suggested that feeding practices could be the most influential factor in shaping CEBs, and that altered eating behaviors can be due to feeding practices that have overridden the child’s ability to self-regulate food intake [15, 16]. Specifically, the existing data suggest that parents may alter their feeding practices in response to perceptions towards the eating behavior traits exhibited among their children [31, 32] and concerns for children’s weight status [33]. A previous study investigated the association of maternal concern for child’s overweight status with the feeding practices; Mothers with greater concern were less likely to use pressure to eat and more likely to restrict food; However, maternal feeding practices and children’s overweight status were not found to be associated [34]. Nevertheless, prospective studies are needed to elucidate whether maternal concern about child weight would result in higher weight status on the long-term.

A previous study conducted among Australian children and their mothers observed greater use of restriction among mothers with higher levels of concern for their child being overweight, whereas no association was observed with maternal use of food as a reward or monitoring feeding practices [10]. Similar finding has been reported by a study conducted in the Unites States [13]. Contrary to these findings, our data did not indicate an association between maternal concern towards child body weight and maternal feeding practices, including food restriction, monitoring, and using food as a reward. This variation could be attributed to cross-cultural differences in societal norms for the role and responsibility of the Saudi mothers in child feeding along with the accessibility to childcare centers, which may affect the quality time spent between the mother and her child [20, 35].

Slowness in eating has been linked to decreased energy intake [6], whereas increased eating speed was found to be associated with child obesity [7]. In the present study, BMI-z score of children was negatively correlated with slowness in eating. Hence, children may benefit from utilizing a family-based intervention approach targeting to reduce speed of eating. A study conducted by Faith et al. has examined the effectiveness of “Reduced Eating Pace” intervention program that aimed to improve CEBs. Findings indicated that the use of psychoeducational and behavioral techniques resulted in increased slowness in eating and food enjoyment and less weight gain [36].

Our data showed a negative association between maternal perception about the child overweight risk and child slowness in eating, whereas no association between maternal perception and maternal feeding practices was observed. These findings suggest that children of mothers who perceive the child as being overweight or obese are possibly reacting to maternal perception. Perhaps, the child is translating his/her emotions by reacting negatively to maternal concern, increasing rate of eating to rapidly finish the served meal. However, further studies are needed to understand factors that could influence child’s eating speed.

To the best of our knowledge, this study was the first to evaluate the associations between maternal concern and perception about child overweight risk and CEBs. Anthropometric measurements of children were objectively assessed, and the sample was recruited from several preschools located in various regions around Jeddah city, Saudi Arabia. Limitations of our study include its relatively small sample size and the low response rate. Incentivizing research participants in future work might be necessary to encourage participation and to enhance response rates [37]. Furthermore, maternal height and weight were self-reported, which could be susceptible to recall bias. Additionally, due to the high proportion of mothers holding a college degree, findings of this study might not be generalizable to children of mothers with lower educational status.

This study provides evidence that maternal concern and perception about child overweight risk are linked to obesogenic CEBs. Specifically, maternal concern about child weight was found to be associated with higher levels of child enjoyment and responsiveness to food and lower levels of food fussiness, whereas maternal perception about child overweight risk was negatively associated with child slowness in eating. Our findings will inform childhood obesity interventions targeting CEBs to address maternal concern and perception about child weight status and to incorporate mothers into the intervention programs. Mothers who are concerned about their child weight or perceive their children as overweight may need guidance and support to promote healthy CEBs. Furthermore, and in contrast to findings of other studies, we did not detect an association between maternal concern or perception about child overweight risk and maternal feeding practices. This may indicate the variation in the expression or extent of maternal concerns across cultures. Future qualitative cross-cultural studies are needed to understand the underlying mechanisms for the influence of maternal concern and perception about child overweight risk on CEBs and weight status. Additionally, further longitudinal research studies may examine the cause-and-effect relationships between maternal concern and perception about child overweight risk and CEBs.

## Supporting information

S1 File (XLSX)
